# Inheritance of *Solanum* chloroplast genomic DNA in interspecific hybrids

**DOI:** 10.1080/23802359.2020.1866450

**Published:** 2021-02-08

**Authors:** Dandan Li, Guiyun Gan, Weiliu Li, Wenjia Li, Yaqin Jiang, Xuyu Liang, Ning Yu, Riyuan Chen, Yikui Wang

**Affiliations:** a College of Horticulture, South China Agricultural University, Guangzhou, China; b Institute of Vegetable Research, Guangxi Academy of Agricultural Sciences, Nanning, China

**Keywords:** *Solanum*, chloroplast genome, interspecific hybrid, inheritance, maternal

## Abstract

The chloroplast genomic information was obtained from three wild *Solanum* and four hybrids by chloroplast genome sequencing. The chloroplast genomes of the seven samples comprise of a circular structure and sizes from 155,581 to 155,612 bp and composed of 130 genes. The genome structures of the two hybrids were identical, while the other two hybrids showed 2 bp differences in the LSC when compared with their maternal parent. The total sites of SNP and InDel were 39–344 and 54–90, respectively. With the exception of one hybrid with two additional sites, the other hybrids were identical to their maternal.

## 1. Introduction

*Solanum* L. is a rich genus of plants comprising ∼1400 diverse species, as one of the largest flowering plant genera (Bentolila and Stefanov 2012; Lim 2013). *Solanum* species are worldwide, including Antarctica, with wild resources found across southeast Asia, Africa, South America, and south China (Giacomin et al. 2013). Wild *Solanum* has several beneficial properties, including resistant and strong growth characteristics (Christodoulakis et al. 2009; Meyer et al. 2012; Rotino et al. 2014). In particular, the aubergine *Solanum melongena* harbors numerous genes that are beneficial for long-term breeding processes (Augustinos et al. 2016), which can be introduced to wild resource species through crossing to broaden the genetic basis as an important means of crop improvement and germplasm innovation. To date, *S. melongena* has been hybridized with *Solanum aethiopicum*, *Solanum anthropophagoru*, *Solanum sysimbriifolium*, and other closely related wild relatives of *Leptostemonum*, showing a significantly higher affinity than other species such as *Solanum pecteinocarpum* and *Solanum aviculare* (Poczai et al. 2008; Frary et al. 2011; Plazas et al. 2016).

Studies on the distant hybridization of *Solanum* have shown that the traits of hybrid offspring were more similar to those of wild resources with strong heterosis (Rotino et al. 2014; Kaushik et al. 2016). Although the growth and development advantages of distant hybrid offspring are known to be correlated with photosynthesis, most of the research on heterosis conducted to date has mainly focused on nuclear gene expression and regulation (Birchler et al. 2010; Meyer et al. 2010; Birchler et al. 2016; Jensen et al. 2018; Wang et al. 2018) and there is no information available as to whether chloroplasts play a role in *Solanum* heterosis. Heterosis is now widely used in the commercial production of many crop hybrids. For hybrid seed production, a controlled pollination system should be developed to prevent unnecessary self-pollination. Hence, male sterility plays an important role in heterosis utilization. It is of great theoretical and practical significance to explore and utilize the male-sterile new germplasm of plants so as to prevent an epidemic of diseases and insect pests in heterosis and promote yield improvements. Most *Solanum* interspecific hybrids are sterile (Afful et al. 2018), which provide precious materials to use and study. Further, most researchers think male sterility results from disturbed mitochondrial–nuclear interaction (Bentolila and Stefanov 2012; Liu et al. 2013), and some have reported the effects of chloroplast genome on plant male sterility (Liu 1983; He 2003).

The chloroplast is a plastid, which is a semi-autonomous plant organelle containing an independent genome (i.e. plastid DNA) with a length between 120 and 230 kb that encodes 100–130 proteins in higher plants. The transcription and translation of plastid genes have many conservative prokaryotic characteristics (Bock 2007; Alice 2011), and chloroplasts harbor the complete enzymes required for photosynthetic substrates and electron carriers in the thylakoid membranes. Chloroplast genome analyses have revealed two main modes of plastid inheritance in angiosperms: biparental inheritance and uniparental inheritance. Maternal inheritance is most common in angiosperms (Xiaohua et al. 2003; Jansen and Ruhlman 2012; Li et al. 2013), with only a few plastids being regularly or occasionally inherited in a biparental manner (Corriveau and Coleman 1988), whereas paternal plastids are more common in gymnosperms (Shiyi 1997), such as *Medicago sativa* (Diatchenko et al. 1996; Pellet et al. 1996), *Daucus* L (Iturbe-Ormaetxe et al. 2003), and *Pharbitis* (Hayashi et al. 2004). The cytoplasmic inheritance of *Solanum tuberosum* is maternal (Hu et al. 2008), which results from the decay of the male organelles themselves or the degradation of DNA, leading to the degradation and disappearance of plastids during the maturation process (Hagemann and Schröder 1989).

The chloroplast is an important photosynthetic organ in higher plants, and has a close relationship with major agricultural aspects, such as crop yield, tinea genetic, cytoplasmic male sterility, photosynthetic efficiency; thus, the deep study of higher plant chloroplast genetics is necessary. To determine the precise genetic pattern of plant chloroplasts is of great significance for phylogeny, biogeography, hybridization, and systematics.

Therefore, we investigated both the intraspecific and interspecific genetic patterns of chloroplast genomes among different hybrid combinations of *Solanum* and further explored the relationship between heterosis, male sterility, and chloroplast genomic effects. Specifically, we assembled the chloroplast genomes of three close self-crossing species of *Solanum* and their four interspecific hybrid F1 lines. These results can provide valuable baseline information on the genetic pattern of chloroplasts in the interspecific hybridization of *Solanum*.

## 2. Results

### 2.1. Genome assembly and genetic features

In total, 134,071 Mb of raw data were obtained from the seven species on the Illumina HiSeq4000 and Pabico platforms. From each species, 4317–6129 Mb of clean data were used to assemble the complete chloroplast genome of *Solanum*. The chloroplast genome comprises a double-stranded circular DNA sequence with a length of 155,581–155,612 bp. The chloroplast genome consists of a large single-copy (LSC) region of 86,160–86,196 bp, small single-copy (SSC) region of 18,501–18,562 bp, and two inverse repeat (IR) regions, IRA, and IRB, of 25,440–25,443 bp each. The GC contents of the seven chloroplast genomes were similar at 37.7–37.71%, which is in line with that previously reported for *S. melongena* (Ding et al. 2016).

The chloroplast genome structure of the hybrids *S. melongena* (177) × *S. aethiopicum* (y11) and *S. aethiopicum* (53) × *S. aethiopicum* (y11) was identical to that of their respective maternal genomes, whereas *S. melongena* (177) × *S. torvum* showed a 2-bp difference and *S. aethiopicum* (y11) × *S. melongena* (177) had a 4-bp, 1-bp, and 2-bp reduction in the LSC, IR, and SSC, respectively, relative to the maternal plant; all hybrids had GC content identical to that of the maternal plant (Table 1).

**Table 1.** Comparison of chloroplast sequences of the seven samples.

Sample	Species	Total reads	Average organelle depth	Total length (bp)	GC content (%)	LSC length	IR length (bp)	SSC length (bp)
177	*S. melongena*	60,736,270	1521	155,581	37.71%	86,194	25,443	18,501
177 × y11	S. *melongena*×S. *aethiopicum*	42,488,436	1824	155,581	37.71%	86,194	25,443	18,501
177 ×*torvum*	*S. melongena × S. torvum*	43,425,370	1599	155,583	37.71%	86,196	25,443	18,501
53	*S. aethiopicum*	62,578,308	1288	155,598	37.70%	86,160	25,440	18,558
53 × y11	*S. aethiopicum × S. aethiopicm*	37,064,318	1722	155,598	37.70%	86,160	25,440	18,558
y11	*S. aethiopicum*	32,820,146	991	155,614	37.70%	86,168	25,441	18,564
y11 × 177	*S. aethiopicum × S. melongena*	34,130,550	764	155,606	37.70%	86,164	25,440	18,562

**Figure 1.**
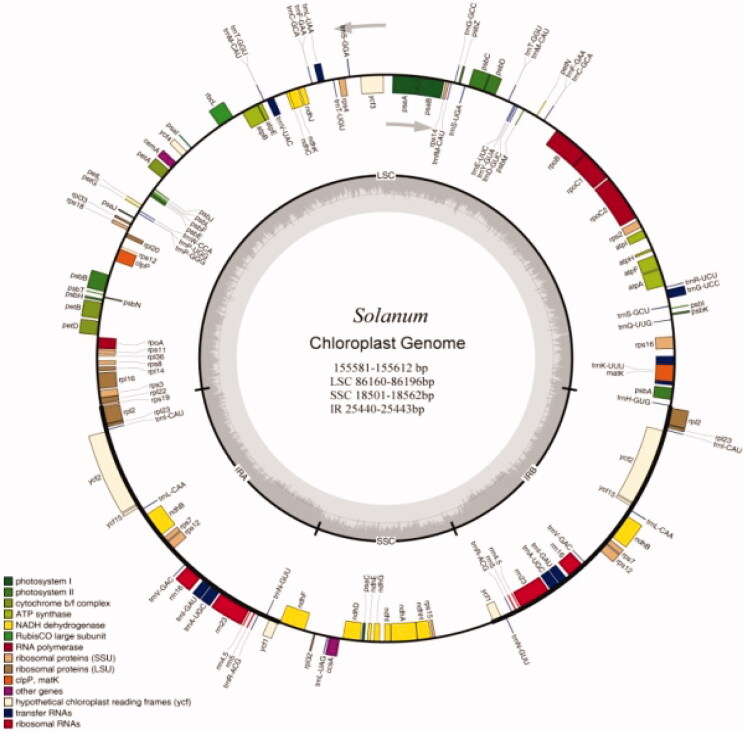
Chloroplast reference genome of seven *Solanum* species. Genes shown inside the outer circle are transcribed counter clockwise and those outside are transcribed clockwise. Genes belonging to different functional groups are color-coded. The gray area in the inner circle indicates the GC content (see Tables 2 and 3 for sample details).

The *Solanum* chloroplast genome contains 130 (106 unique) functional genes, including 80 (71 unique plus *ycf* (#1, #2, #15) as hypotheticals) protein-coding genes, 42 (31 unique) tRNA genes, and eight (four unique) rRNA genes. Furthermore, 17 genes were found to be replicated in both IR regions, including six protein-coding genes and 11 RNA genes (four rRNAs and seven tRNAs; Table 3). The LSC region contains 60 protein-coding and 27 tRNA genes, while the SSC region has 11 protein-coding genes and one tRNA gene (Table 2 and Figure 1).

**Table 2.** List of genes encoded in the chloroplast genome of the seven *Solanum* species.

Function	Gene group	Gene name
Photosynthesis pathways	Photosystem I	psa (A, B, C*,I, J)
	Photosystem I assembly	*ycf (3, 4)*
	Photosystem II	*psb (A–F, H–L, N, T, Z)*
	F-type ATP synthase	*atp (A, B, E, F, H, I)*
	NAD(P)H-dehydrogenase complex	ndh (A*, B#, C, D*, E*, F*, G*, H*, I*, J, K)
	Component of cytochrome b6/f complex	*pet (A, B, D, L)*
	Inner envelope membrane	*cemA*
	Cytochrome biogenesis protein	ccsA*
	Large subunit of Rubisco	*rbcL*
Structural RNAs	Transfer RNAs	*trnH-GUG; trnK-UUU; trnQ-UUG; trnS-GCU; trnG-UCC; trnR-UCU; trnC-GCA; trnF-GAA; trnD-GUC; trnY-GUA; trnE-UUC; trnT-GGU; trnM-CAU; trnS-UGA; trnG-GCC; trnfM-CAU; trnS-GGA; trnT-UGU; trnL-UAA; trnV-UAC; trnW-CCA; trnP-UGG; trnP-GGG; trnI-GAU#; trnA-UGC#; trnN-GUU#; trnL-UAG; trnR-ACG#; trnV-GAC#; trnL-CAA#; trnI-CAU#*
	Ribosomal RNAs	*rrn (4.5#, 5#, 16#, 23#)*
Genetic apparatus	Large subunit of ribosomal protein	rpl (2#, 14, 16, 20, 22, 23#, 32*, 33, 36)

**Table 3.** SNP types of the chloroplast genomes of the seven *Solanum* species.

Sample	Stop_Nonsynonymous	Synonymous	Nonsynonymous	Total_CDS_SNP	Intergenic	Total_SNP
177	1	1	7	9	30	39
177 × y11	1	1	7	9	30	39
177 ×*torvum*	1	1	7	9	30	39
53	1	59	40	100	239	339
53 × y11	1	59	40	100	239	339
y11	1	57	41	99	243	342
y11 × 177	1	58	41	100	244	344

### 2.2. Comparative genome analysis

Using KU682719 (*S. melongena* plastid) as the reference, an analysis of the single nucleotide polymorphisms (SNPs) of the seven chloroplast genomes (Table 3) showed that the hybrid *S. aethiopicum* (y11) × *S. melongena* (177) had one additional synonymous substitution in the coding sequence area and intergenic area, respectively. However, the other three hybrids were completely consistent with their maternal plants with respect to the quantity, type, and location of SNPs, with vast differences observed between different female parents. There was a total of 39 SNP sites in 177 (*S. melongena*) and its two hybrids, 76.9% of which were in the intergenic region. *S. aethiopicum* 53 and y11 had 339 and 342 SNPs, respectively, with few differences between them, and 71% of the SNP sites were in the intergenic region.

Using KU682719 (*S. melongena* plastid) as the reference, an analysis of the insertions/deletions (InDels) of the seven chloroplast genomes (Table 4) showed that *S. aethiopicum* (y11) × *S. melongena* (177) has two additional loci in the intergenic region, and the other three hybrids were completely consistent with their maternal plants with regards to quantity, type, and location of InDels, with large differences among the different female parents. There were 54 InDel sites of 177 (*S. melongena*) and its two hybrids (177 as the maternal plant), 85.2% of which were in the intergenic region. *S. aethiopicum* 53 and y11 had 88–92 InDels, with minimal difference between them, and 63% of the InDel sites were in the intergenic region. The predominant InDel type was insertions, accounting for more than 63% of all InDels identified.

**Table 4.** InDel types of the chloroplast genomes of the seven *Solanum* species.

Sample ID	I_gene_start	I_gene_middle	D_gene_middle	Total_CDS_InDel	Intergenic	Insertion	Deletion	Total
177	0	6	2	8	46	46	8	54
177 × y11	0	6	2	8	46	46	8	54
177 ×torvum	0	6	2	8	46	46	8	54
53	0	4	3	6	86	58	34	92
53 × y11	0	4	3	6	86	58	34	92
y11	1	4	3	7	81	56	32	88
y11 × 177	1	4	3	7	83	57	33	90

### 2.3. Phylogenetic analysis

**Figure 2.**
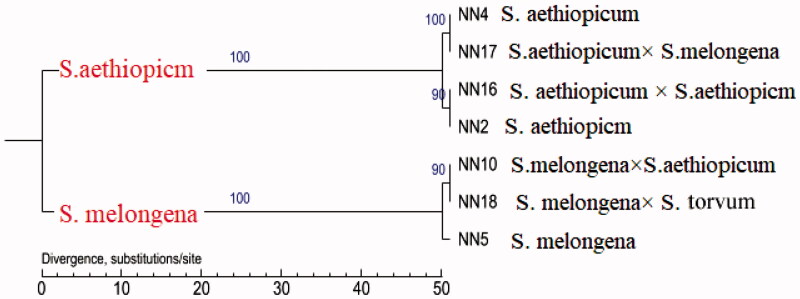
Phylogenetic analysis of the seven *Solanum* species.

The assembled NN2 chloroplast genome was used as the reference genome for the construction of the phylogenetic tree based on the SNP data of the chloroplast genomes of the seven sequenced materials. As shown in Figure 2, the phylogenetic tree was mainly divided into two branches: one branch made up of *S. melongena* and its hybrids *S. melongena* × *S. aethiopicum* and *S. melongena* × *S. torvum*, and the other made up of *S. aethiopicum* and its hybrids *S. aethiopicum* × *S. melongena* and *S. aethiopicum* × *S. aethiopicum*. This result indicates that the chloroplast of *Solanum* is maternally inherited.

## 3. Discussion

The cytoplasmic genetic patterns of more than 600 species of angiosperms have been identified to date (Nagata 2010). The most common methods currently used to identify the genetic pattern of chloroplasts are cytogenetics, use of fluorescent dyes (Quan and Yang 2003), molecular biology approaches (Testolin and Cipriani 1997; Xiaohua et al. 2003; Hansen et al. 2007; Zora and Pal 2007), and direct high-throughput sequencing. However, it is difficult to identify spermatozoa from the ultrastructure because of only a few morphological differences between male and female gametes, especially for plastids that are closely related. Fluorescence staining is a simple, sensitive, and rapid technique, which can directly detect plastids or mitochondria; however, it is difficult to distinguish between plastids and mitochondria with this approach. Molecular biology methods, including Southern hybridization or random primer amplification, can be directly used to determine the presence and transmission of plastids or mitochondria; however, these methods are hindered by the limited number of available probes or primers, making it difficult to determine the genetic status of organelles accurately and comprehensively. With the development of next-generation sequencing technology, a large amount of chloroplast sequence data can be obtained quickly and in a cost-effective manner, which is also the most direct method to determine the genetic mode of inheritance.

The genome structure, GC content, and annotated genes, in addition to the quantity, type, and location of SNPs and InDels of the hybrids are nearly the same as those in the maternal plant, which showed that the *Solanum* genus presents the typical pattern of matrilineal chloroplast inheritance. This has important implications for *Solanum* speciation because isolation and differentiation are the basic mechanisms of new species formation; however, there is no strict reproductive isolation between *Solanum* species, and the pollen has a certain interspecies affinity, which results in a wide variety of hybrid materials and local varieties that can be produced. Consequently, there is substantial debate regarding the evolutionary relationships and classifications of these intermediate hybrid materials and wild species. The present study confirmed that the *Solanum* genus shows the typical pattern of matrilineal chloroplast inheritance. Although different species could hybridize, they cannot form a new taxon.

Furthermore, the distant hybrids between *Solanum* species have excellent characteristics, including improved stress resistance (Collonnier et al. 2003; Iwamoto et al. 2007; Zhou et al. 2018), growth, and development (unpublished). However, hybrids share the same chloroplast system as their maternal counterparts. The hybrid formation process does not involve the recombination of chloroplast genomes of both parents, which means that heterosis has no relation with the chloroplast genome, but rather results from nuclear gene interactions that require further research.

In particular, CMS in plants is mainly caused by the nucleus of a species being replaced with a different cytoplasm, and many CMS materials are obtained through interspecific or intergeneric hybridization. In this study, three interspecific hybrids were sterile, which likely resulted from disturbed mitochondrial–nuclear interactions rather than the effects of the chloroplast genome. Our study shows that sterile F1s possess the same chloroplast genome similar to those of their maternal counterparts; therefore, the *Solanum* male sterility observed in this study is unrelated to the chloroplast genome.

## 4. Materials and methods

The self-cross-breeding materials of three Solanum species and the four hybrids were obtained from the Vegetable Research Institute seed bank, Guangxi Academy of Agricultural Science (28°N and 118°E) (Table 5).

**Table 5.** Basic information of the seven *Solanum* plants.

Sample ID	Code	Genome accession number	The original data accession number
NN2	53	MN218077	SAMN16746488
NN4	Y11	MN218079	SAMN16746490
NN5	177	MN218080	SAMN16746491
NN10	177 × y11	MN218085	SAMN16746496
NN17	y11 × 177	MN218092	SAMN16746503
NN16	53 × y11	MN218091	SAMN16746502
NN18	177 × torvum	MN218093	SAMN16746504

### 4.1. DNA extraction, genome sequencing, and assembly

Approximately 5 g of fresh leaves were harvested for cpDNA isolation, using an improved extraction method (McPherson et al. 2013). After DNA isolation, 1 μg of purified DNA was fragmented to construct short-insert libraries (insert size 430 bp) according to the manufacturer’s instructions (Illumina), followed by sequencing on the Illumina HiSeq 4000 (Borgström et al. 2011). The high molecular weight DNA was purified and used for PacBio library preparation, Blue Pippin size selection, and then sequenced on the Sequel Sequencer. Prior to assembly, Illumina raw reads were filtered. This filtering step was performed to remove the reads with adaptors, reads showing a quality score below 20 (*Q* < 20), reads containing a percentage of uncalled based (‘*N*’ characters) equal or greater than 10%, and duplicated sequences. The chloroplast genome was reconstructed using a combination of PacBio Sequel data and Illumina HiSeq data, and the following three steps were used to assemble the cp genomes (Richard 2008). First, the genome framework was assembled using both Illumina and PacBio data by SPAdes v3.10.1 (Antipov et al. 2016). Second, the assembly was verified, and the circle characteristics of the cp genomes were completed, filling any gaps. Third, clean reads were mapped to the assembled cp genome to correct the incorrect bases, and insertion and deletion were assessed.

### Genome annotation

The chloroplast genes were annotated using the online DOGMA tool (Wyman et al. 2004), using default parameters to predict protein-coding genes, tRNA genes, and rRNA genes. A whole chloroplast genome Blast (Lobo 2012) search (*E*-value ≤10-5, minimal alignment length percentage ≥40%) was performed against five databases: KEGG (Kaneisha et al. 2004, 2006), COG (Tatusov et al. 1997, 2003), NR, Swiss-Pro (Magrane 2011), and GO (Ashburner et al. 2000). The circular Solanum chloroplast genome map was drawn using Organellar Genome DRAW v1.2 (Lohse et al. 2007).

The cp genome among 13 species was compared using the VISTA program. Genome, protein-coding gene, intron, and spacer sequence divergences were evaluated using DnaSP 5.10 after alignment. The genomic sequences were aligned using MAFFT v5, and were adjusted manually where necessary. For the protein-coding gene sequences, introns, and spacers, every gene or fragment was edited using the ClustalW multiple alignment option within the software BioEdit v7.0.9.0.

### 4.2. Comparative genome analysis

MUMmer and BLAST were used to conduct global alignment and local alignment between the sample sequence and the reference genome, determining potential SNPs. Subsequently, the SNPs were filtered out in the repeat regions as detected using the software BLAST, Repeat Masker, and TRF. Finally, SNPs were annotated based on the position and interaction between genes.

LASTZ software was also used to perform global alignment between each sample sequence and the reference genome. Subsequently, the alignment result was corrected by axt_correction, axtSort, and axtBest to determine potential InDels with lengths less than 50 bp. Finally, BWA and SAM tools were used to map the reads to InDel sequences and to filter out unreliable InDels.

### 4.3. Phylogenetic analysis

ClustalW was used to align the cpDNA sequences under default parameters (Larkin et al. 2007), and the alignment was checked manually. The maximum-likelihood (ML) methods were performed for genome-wide phylogenetic analyses using PhyML 3.0 (Guindon et al. 2010). Selection of the nucleotide substitution model was done using jModelTest 2.1.10 (Darriba et al. 2012) and Smart Model Selection in PhyML 3.0. The model GTR + G was selected for ML analyses with 1,000 bootstrap replicates to calculate the bootstrap values (BS) of the topology. The results were treated with iTOL 3.4.3 (Letunic and Bork 2016).

## 5. Conclusions

In this study, we used a high-throughput sequencing approach to characterize the chloroplast genome of *Solanum*, including three wild species and four hybrids to determine the mode of inheritance, extent of heterosis through hybridization, phylogenetic relationships, and the relationship between the male stile and chloroplast genome. We believe that our study makes a significant contribution to the literature because appropriate cross-breeding can be used to obtain hybrid varieties with excellent characteristics for crops, including improved growth and stress resistance, particularly for aubergine (*S. melongena*) as an important *Solanum* crop worldwide. Therefore, understanding the contribution of cytoplasmic inheritance and the chloroplast genome to these traits can aid in obtaining useful germplasm. Moreover, our results provide further information on chloroplast inheritance toward gaining a better fundamental understanding and facilitating further research on the interspecific heterosis, male sterility mechanism, and evolution of *Solanum*.

## Data Availability

The raw data that support the findings of this study are openly available in GenBank of NCBI at https://www.ncbi.nlm.nih.gov, reference numbers: SAMN16746488, SAMN16746490, SAMN16746491, SAMN16746496, SAMN16746503, SAMN16746502, and SAMN16746504.

## Abbreviations

BS: bootstrap
CML: cytoplasmic male sterility
COG: clusters of orthologous groups
cpDNA: chloroplast DNA
GO: gene ontology
KEGG: Kyoto Encyclopedia of Genes and Genomes
InDel: insertion/deletions
LSC: large single-copy
NR: Non-Redundant Protein Database
SNP: short nucleotide polymorphism
tRNA: transfer RNA
